# Success in Life

**Published:** 1878-07

**Authors:** Ausburn Towner


### SUCCESS IN LIFE.

WITH A PERSONAL APPLICATION.*

BY AUSBURN TOWNER.

The world of boyhood or school-days is quite separate and distinct by itself, seldom, if ever, reaching over, except as regards certain well-defined prejudices, into the world that follows or comes with manhood. What man is there who has not had the experience of the first shock that the sight of one certain little girl gave him when he was in roundabouts? She was a fairy, a sylph, a being so spiritual and almost unreal, that you hardly dared to touch her lest she disappear like a breath. You may have seen her first, at school; in the woods picking berries, or at church. She may have been clothed in calico, with a sun bonnet so big that its ruffle fell to her knees and obscured her face, or she may have been decked out in all the radiance of bright ribbons, and the gayest of gay attire. It was all the same. The shock came, and though you could not tell what it was or what it meant, it was strong and never quite obliterated. When you touched her hand, your blood ran swifter through your veins, and when she spoke, it was music. Perhaps she died, and your last recollection of her is of one lying calm and still in white robes, almost hidden among the flowers. And often now, wrinkled and gray-haired, with some one else sitting opposite you at your table, you think of her and say to yourself, "Oh if—." And the "if" carries you through an infinite number of possibilities, none of which might have been at all possible.

But, ten to one, she didn't die, and as you grow older, the youthful film gradually comes away from your eyes, and when you see her, as she has become "Mrs. Something-or-other Rogers," you daily grow more and more astonished that you could ever have surrounded her with such a halo as you once manufactured out of whole cloth. Keep on being astonished. It is only fair. Rest in the assurance that in her mind are passing similar thoughts in regard to yourself. Rest in the assurance that she, too, is saying to herself: "Is it

*PREFACE. Not in the way of excuse or apology for what is to follow, but clearing the way before hand. The editorial paragraph in the last BISTOURY, referring to my contribution in that number, aroused a flood of recollections and memories that for many days has held the chief place in my mind, during my leisure hours. The putting them upon paper, it seems to me, would have a similar agreeable effect upon the large number of readers and admirers of the BISTOURY, who will recognize the personal allusions herein, and to the general reader, they can but bring somewhat similar recollections even if about far different persons.

possible that that care-worn, bald-headed, wrinkled, absent-minded man seemed once so brave and thoughtful and rosy to me!"

To a greater degree as between boy and boy is this feeling of semi-worship built up, for the difference in sex does not operate for many years yet. If you would know of it in its maximum, you have but to recall or to read the account of the school-days of David Copperfield as related by the great master of the human heart and human character. Every boy has his Steerforth. There is some lad older or of the same age whom his consciousness instinctively acknowledges as his superior, little short of his God. It is in the experience of every one that they have seen some little friend, perfectly ungovernable by father or mother, who will implicitly obey the behest of some playmate even into the extremity of personal danger, never once thinking of rebelling against his authority. Who does not remember a lad or friend of his childhood, who seemed to possess all of the graces and excellencies possible to be possessed by human kind? How handsome he was! How you followed him with your eyes in whatever situation he might be placed. How you magnified his successes on the play-ground or in the school-room, and excused his failures. He didn't half try. He was too generous to his opponents. There were prejudices against him or he didn't have half a chance. How you dreamed over his future and prophesied that he would be a leader among his fellow-men as he was among his fellow boys. In ninety-nine cases out of one hundred, time overthrows the idol you have builded up, and when the downfall comes, how sore it makes your heart. Some one the least thought of, some modest, unassuming and frequently lazy and careless lad, girding himself for the real struggle of life, passes and leaves far behind in the contest him whom your young thoughts had set up to be the first at the goal. I well recollect one hero of my childhood, who seemed to me to be always first in everything that he undertook, and I recollect with a shudder even now when he came tumbling down from the position to which I had elevated him. It was when I saw him drunk for the first time. The first time, I say, for his inebriety, alas! has become an old story now. I had been bred to dread a drunken man much more than an insane one. There was a mixture of pity combined with positive fear that I had always had for one completely under the influence of liquor, which it is hard to overcome even now, and to see my idol in such a condition entirely upset him from his place in my estimation. These things come stronger upon one, if the place of his boyhood is removed from the place of his manhood, and the first is not visited for many years. How the glamour and halo of the earlier days is rubbed off by the later, leaving the youthful heroes very commonplace men indeed.

There is something of this in the larger life of men, and those who cannot feel as they feel, are almost amused at the earnestness and zeal with which some men clothe certain other men with extraordinary characteristics. Thus Addison writing of Milton, James T. Fields of Hawthorne, Col. Higginson of Thoreau, George William Curtis of Thackeray, or the average critic who desires to be thought very judicious but who is only full of cant and commonplace, writing of George Elliot, and lastly Boswell writing of Johnson, are but the natural ebullitions of an intellect seeking something human to admire, and pouring out adoration which seems manly among boys, and boyish among men. You say with an advance in years one should recover from such attacks. Perhaps they should, but in these instances which I have mentioned, the disease, for it is only a disease, has struck in deep.

Now a man's success in life is in the majority of cases, largely due to just this sentiment upon which I have been enlarging. In the brain of some one, the idea is conceived that a certain other one possesses some characteristics that are extraordinary. If this some one is a man of position and influence, the upward path of that certain one is easy and sure, for what that some one says is listened to and believed. And even if that some one is moderately obscure he will find many who will finally believe in whatever reiterated assertion he may make. It is the chorus that makes the solo singers so prominent. "He is an extraordinary man," frequently repeated, will make any one seem so, whether he may be really so or not. And the want of his faithful Achates, the absence of a one-voiced chorus, if the term may be used, is fatal to the hopes and ambitions of any one may he be ever so meritorious or deserving. You may say that the man who pushes himself along gets ahead, that "Whosoever bloweth not his own horn, his horn shall not be blowed." This is not so. The public will not stand for any length of time, even a self-asserting genius whose self-assertion is too patent; the one who tries to get on in a sail boat, by means of wind furnished by a bellows worked by himself; or the other one who tries to lift himself over a fence by the straps of his pantaloons, will find that in the first instance, he goes backward, and in the last, that he is on his back very quickly. The best men everywhere understand this fact if they do not recognize the principle. It extends even from the puddle of local politics to the highest and purest endeavor in which men can be engaged. It won't do for a man to speak for and of himself. Some friend does it for him. Cæsar was three times presented with a crown, and three times surprised (?) at the offer, pushed it away. But back of the offer, hidden away from the apprehension of the popular mind is the hand of Cæsar himself. One can pull the strings of the puppets if he so desires, but the pulling must not be seen.

My reader must doubtless be able to call to mind at least one instance, some of them may be able to recollect many, of persons whom they think if there had been some one to hold up their hands for a brief time in the beginning until the world could see the work of which they were capable, would have been appreciated, and would have achieved their places in the world to which their merits entitled them, but who, there being no one to turn upon them the strong rays of public attention, passed their lives in shadow and obscurity, recognized by few and loved by the still smaller number who knew and understood them. Whose modesty and unassuming gentleness forbade them from even themselves, putting in train a chain of circumstances that would ultimately result in their being called forth from their retirement; who waited patiently but vainly, working industriously meanwhile to be called, as we are told were the men of God, of old.

I have in my mind one, and there are very many amongst us who will, as well, remember him to whom I allude, and will also acknowledge the truthfulness and justness of the words that I would say of him. He was mentioned by name in the last number of the BISTOURY; Will C. Potter. These words come many months after he has escaped forever from the many sore trials and disappointments that he suffered in this life, but they are nevertheless sincere, the only regret in the midst of them being, that he cannot receive the benefit of them, whatever that benefit might be. I do not forget too, that there are some, not many, thank Heaven! who will say "His work was below the average; what he did was common, else why was he not more known?" And in most instances, these who thus speak, while they don't know what they are talking about, would have been the first to flatter, had the sun ever shone upon him.

The chief motive of Potter's life, seemed to those who knew him best, to be that exalted one which submerges self entirely, and is expressed in the words, "What can I do for some one?" He has often been accused of continually asking himself that question. There is a little illustration of it, in his younger days. There was a debating society amongst the lads in attendance upon the old District School on William street, which used to stand where the Sheriff's residence is now located. I wish I could invade private life so much as to nominate some of those who were members of this organization. They are prominent men; prosperous men. Merchants, lawyers, doctors and bankers, now in our midst. The subject one night was a trite one then, as to our feelings towards the negroes at the South, the Slaves; and the American Indians. Whether or not it was a new view of the subject, I cannot tell, but Potter's theory struck us all as peculiar and carried the day or night over all opposition. It was, that the Indians were superior to the blacks and for them he could have sympathy; for the blacks, only pity. Sympathy being the more elevated sentiment. It was a little thing, even for a lad, but was in the line of his deeper feelings and his amplification of his theme, even to a mature intellect, is recalled with something more than mere satisfaction.

He had a taste for the finer and more delicate things of this life; an eye for color and harmony; a vivid sense of form, and took intense delight in the highest qualities of music and literature. It was quite natural that he should then seek to become a painter. What he knew and became in this, he knew and became by his own, unaided efforts. Many things of detail and purely mechanical skill, that a master in one year's time could have thoroughly taught him, he discovered himself, as though he had been the first to use them. In the which, he deserved, morally, as much credit as though he really *had* been the first, but of course he couldn't get it for the path was well trodden beforehand. He was like one who, from mere lack of means, but anxious and determined to go to New York—should start off on foot. His friend, nowise his superior, save in the possession of means, jumps on the cars, and is whirled to the same place in a night. Weeks pass, and our foot passenger, tired, sore, and lame, also arrives at the spot where he so longed to be. Which journey is the harder, and which one deserves the most credit for making it? Nevertheless he who goes by the train is far in advance of his brother, and reaps the larger reward, always.

The skill that Potter eventually obtained in putting scenes of nature upon the canvass was quite equalled by his descriptions in words of that which he saw. His habits of observation were marvelous, and in the twilight of his own studio it was a great treat to hear him tell of a walk he had taken about the hills of the Chemung, or of some incident he had witnessed. There was one point in this locality which his imagination often turned upon, and the correctness of which will be seen by any one who is familiar with the scenes alluded to. It will repay any one to see for himself.

Standing on East Hill, by the Water Cure, and looking west, you can see where the hills divide to let the Chemung river pass through. On each side they come close down to the water, those on the south almost forming a precipice. Early in June the view is very beautiful, and it is especially so if there is a little mist thereabouts, to allow the fancy to work freely with the surroundings. It is a copy, in Nature, of the famous and romantic spot in Spain known as "the Last Sigh of the Moor." The place where the Moors made their last stand against the advancing and victorious arms of the Christians, where they took their final adieu with sobs and sighs of their beloved Granada. There is a famous painting of this scene, and it requires but little of one's fancy to see how near it is to the view on the Chemung, alluded to.

The limits of a BISTOURY contribution would not allow of a tithe of the recollections that spring to my mind concerning him of whom I speak. All about this part of the country and in some of the best houses in New York, and other large cities, there are hung in honored and conspicuous places, specimens of his work that bear no unfavorable comparison with comrades whose chiefest excellence lies in the fact that in one corner or upon the back is placed the name of one who happens to have gained a prominence and a reputation, whether he earned it or not. A careless piece of veneering with Turner, or Church, or Bierstadt on the frame, will draw an admiring crowd of eyes, while a conscientious, careful production with only Potter there, is passed by. Catch the eye or ear of the world, be your colors never so gaudy, worthless and flaunting, or your words the merest gibberish, and you can hold on while you live.

One more reference. The citizens of Elmira will surely recollect the truly dramatic ability manifested by Potter some twelve or fifteen years ago, in a series of representations of which he was the life and soul, given in what was then Ely Hall, and for the benefit of himself? No. Of the Soldiers' Home, then located on Third street, and carried on under the auspices of loyal and philanthropic ladies. He gathered about himself some of the very best young people of our city; gave his time and services, and during the season, some of the standard and most popular dramas were played to crowded houses, giving to the Home the splendid sum of nearly two thousand dollars! The characters he himself undertook, made manifest to his friends, that if he had chosen the dramatic for his profession, he might, unless pursued by the same ill-fate that clung to him in other walks, have secured prosperity.

There could be no more pleasing task that I could set for myself than to extend indefinitely, reminiscences, incidents and sayings of one who deserved the kindly consideration of the world, quite as much as some others who have received it in full measure; and in calling him to mind, I cannot conclude with a more apt expression than one uttered by a singular character who at one time was as well known in Elmira as is the sound of the fire bell now. He was commonly known as "Bill Dingeldy," and he referred to the one of whom I have been speaking. "Bill" had a big heart, whatever other faults may have possessed him. He was looking at a picture that Potter had just finished; a picture that had been the admiration of our citizens; had been exhibited at the Academy in New York, but which the artist out of the most stringent want was compelled to sell for a mere pittance. "Bill" looked at it long, earnestly and silently, and he knew what a good picture was. Finally, rising to go, with tears of sympathy in his eyes, and with no thought of anything, save his own sincerity, he said:

"Potter, I wish I could be the Almighty for only twenty-four hours. Things don't seem to go right in this world. That's all the time I should want. But I'd be devilish busy, every minute. And the man that could paint such a picture as that, wouldn't be wondering and nervous about where his next meal was to come from."

